# RNAi-mediated gene silencing in tick synganglia: A proof of concept study

**DOI:** 10.1186/1472-6750-8-30

**Published:** 2008-03-26

**Authors:** Shahid Karim, Bronwyn Kenny, Emily Troiano, Thomas N Mather

**Affiliations:** 1 Center for Vector-Borne Disease, University of Rhode Island, 9 East Alumni Ave, Kingston, RI 02881, USA

## Abstract

**Background:**

Progress in generating comprehensive EST libraries and genome sequencing is setting the stage for reverse genetic approaches to gene function studies in the blacklegged tick (*Ixodes scapularis*). However, proving that RNAi can work in nervous tissue has been problematic. Developing an ability to manipulate gene expression in the tick synganglia likely would accelerate understanding of tick neurobiology. Here, we assess gene silencing by RNA interference in the adult female black-legged tick synganglia.

**Results:**

Tick β-Actin and Na^+^-K^+^-ATPase were chosen as targets because both genes express in all tick tissues including synganglia. This allowed us to deliver dsRNA in the unfed adult female ticks and follow a) uptake of dsRNA and b) gene disruption in synganglia. *In vitro *assays demonstrated total disruption of both tick β-Actin and Na^+^-K^+^-ATPase in the synganglia, salivary glands and midguts. When dsRNA was microinjected in unfed adult female ticks, nearly all exhibited target gene disruption in the synganglia once ticks were partially blood fed.

**Conclusion:**

Abdominal injection of dsRNA into unfed adult female ticks appears to silence target gene expression even in the tick synganglia. The ability of dsRNA to cross the blood-brain barrier in ticks suggests that RNAi should prove to be a useful method for dissecting function of synganglia genes expressing specific neuropeptides in order to better assess their role in tick biology.

## Background

Ticks are found in almost every region of the world and are second only to mosquitoes in their public health and veterinary importance [1]. Ticks transmit the greatest variety of pathogens to humans and veterinary species of any arthropod vector [2], including the agents causing Anaplasmosis, Cowdriosis, East Coast Fever, Babesiosis, Lyme borreliosis, tick-borne relapsing fever, ehrlichiosis, Rocky Mountain spotted fever, Boutonneuse fever, Queensland tick typhus, Q fever and numerous arboviruses [3]. Ticks in the *Ixodes persulcatus *complex, including the blacklegged tick, *Ixodes scapularis*, are particularly important vectors of human disease-causing agents; *I. scapularis *ticks can harbor multiple pathogens including the agents of Lyme disease, *Borrelia burgdorferi *[4], human anaplasmosis, *Anaplasma phagocytophilum *[5], human babesiosis, *Babesia microti *[6] and an encephalitis-like virus [7]. *Ixodes *ticks have a life cycle involving egg, larval, nymphal and adult stages. Infected nymphal ticks play the most important role in transmitting disease to humans [8].

Sequencing of the *I. scapularis *genome, along with development of new investigative tools such as expressed sequence tags, microarrays, and RNA interference offer new alternatives for researching tick and tick-borne disease control [9-14]. One very promising method for generating targeted down-regulation of gene expression in a wide range of organisms is RNA interference (RNAi). RNAi is the process by which dsRNA inhibits accumulation of homologous transcripts from cognate genes, thus providing a powerful alternative to more traditional immunization and genetic techniques [15]. RNAi also has evolved into a powerful tool for probing gene function in *Drosophila, Tribolium, Caenorhabditis elegans *and mice [16,17]. Delivery of dsRNA or siRNA into a cell triggers abrogation of the target mRNA. Previous experiments showed the feasibility of using RNAi to abrogate expression of gene transcripts in salivary glands of the ticks, *Amblyomma americanum *and *Ixodes scapularis *[18,12,18-19]. Recently, Hatta et al., [21] demonstrated the RNA interference of cystosolic leucine aminopeptidase not only in the salivary glands, but also in the midgut, ovary and epidermis of *Haemaphysalis longicornis*.

A number of animal cells have been shown to naturally take up exogenous dsRNA and use it to initiate RNAi silencing [22,23]. In some organisms, such as *Drosophila*, certain cells efficiently uptake dsRNA but seem to be unable to transmit this dsRNA to other cells in the fly body [24]. Organisms like *C. elegans *and juvenile grasshoppers can both take up dsRNA and spread it systemically to elicit an RNAi response throughout the entire organism [25,26]. The typical RNAi response occurs in two phases. In the first phase, double-stranded RNAs are cleaved into double stranded fragments called siRNAs by the RNase III enzyme Dicer [27-29]. In the second phase, these siRNAs are conveyed into a large ribonucleoprotein complex known as the RISC [RNA-induced silencing complex] where they act as guides, base-pairing to complementary mRNAs and triggering RISC-mediated mRNA cleavage [27,29,16,33] and destruction [34-36]. The mechanisms of uptake, spreading and processing of dsRNA are poorly understood. Labeled siRNA or dsRNA has been used in tracking of these molecules in mammalian cell lines and in *schistosomes mansoni *[37-39]. Here, we extend the scope of the RNAi technique in *I. scapularis *by describing the uptake and spread of dsRNA in tick synganglia [brain]. To test protocols for gene silencing in tick synganglia, we focused on β-Actin and Na^+^-K^+^-ATPases, genes that plays a conserved role in all tick tissues. We show that gene expression in the synganglia of female adult ticks, as exemplified by the genes coding for the β-Actin and Na^+^-K^+^-ATPase, can be specifically inhibited by microinjection of dsRNA into unfed ticks. In the future, this may make possible the targeting of genes encoding specific neuropeptides expressed only in the synganglia and that play vital roles in tick biology.

## Results

**Figure 1:**
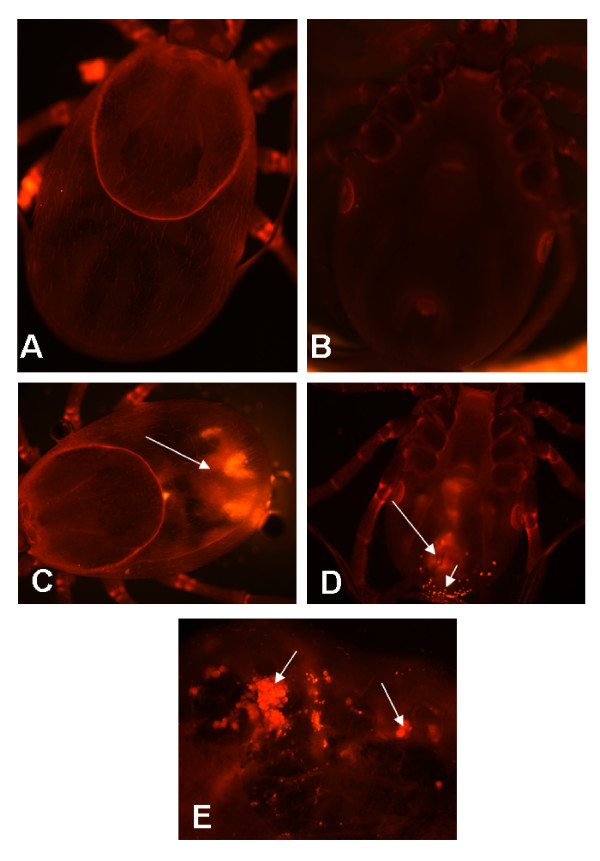
Visualization of Cy3 labeled β-Actin dsRNA in unfed adult female *Ixodes scapularis *ticks: Unfed female adult ticks were injected with Cy3 labeled Actin dsRNA and kept at 37°C overnight. Unfed ticks injected with labeled dsRNA were visualized under confocal microscope. **A-B**: Control ticks injected with Cy3 dye; **C**. Cy3 labeled Actin-dsRNA readily detectable in the unfed tick after injection. Arrow indicates the Cy3 labeled dsRNA; **D) **Labeled dsRNA diffused and degraded after 24 hrs feeding on host. Red-dots on the lower side of the tick may have been excreted labeled dsRNA. Arrow indicates the degradation of labeled dsRNA. **E) **Uptake of Cy3 labeled dsRNA in the dissected salivary glands.

**Figure 2:**
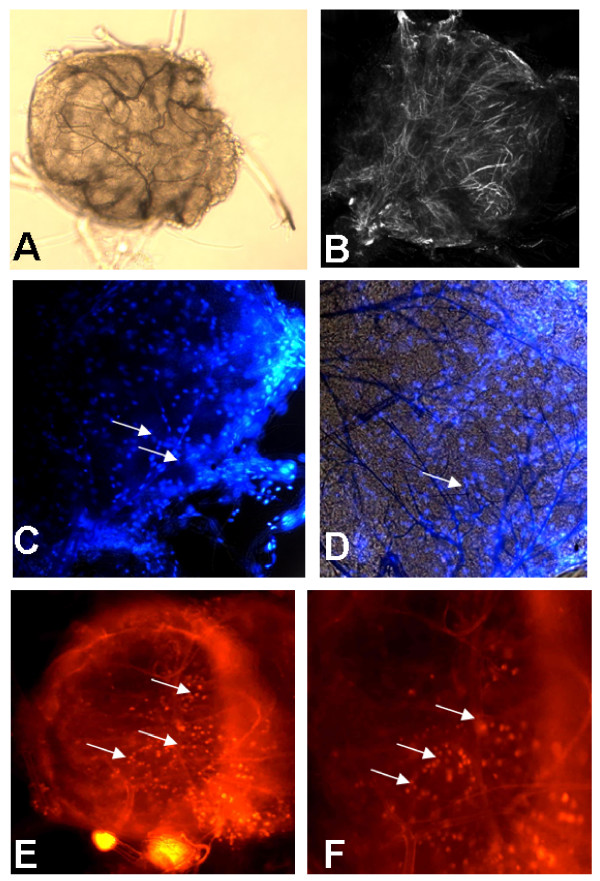
*In vivo *visualization of Cy3 labeled β-Actin dsRNA: Labeled dsRNA was injected in the unfed adult female ticks and fed for 72 hrs on rabbit. 72 hrs fed ticks were dissected and synganglia were removed and visualized under confocal microscopy. **A) **light microscopic image of tick synganglia; **B) **Low infrared image showed a mesh of neurons; **C-D) **tick synganglia stained with DAPI showing neuronal nuclei, and superimposed image (arrows indicate nuclei labeling), **E) **Cy3 labeled dsRNA spread in the whole synganglia (10×) and **F) **40× image (arrows indicate the spread of labeled dsRNA in the synganglia).

One of the major problems in generating dsRNA-mediated gene silencing is delivery of the dsRNA to the target site. Often, distribution of injected dsRNA cannot be visualized, and phenotypes are analyzed in all injected ticks under the assumption that dsRNA are uniformly distributed. To determine whether our dsRNAs became incorporated in tick synganglia and other tissues, we used Cy3 labeled Actin dsRNA. Use of Cy3 labeled dsRNAs permitted monitoring of the uptake of these molecules in tick tissues. Although injections were performed in the tick hemocoel, labeled dsRNA diffused throughout the entire tick (Fig. 1C and 1D). We also observed dotted localization of labeled dsRNA actin in whole adult female ticks (Fig. 1C). This ubiquitous distribution of labeled dsRNAs suggests that dsRNA is rapidly taken up and efficiently distributed throughout the cells in all tick tissues including neuronal cells (Fig. 1D). The accumulation of dotted staining (Fig. 1C) could be a hint that excess labeled dsRNAs can be secreted out by the feeding ticks. The fluorescence also was detected in the tick synganglia at various intensity levels (Fig. 2) indicating that molecules of dsRNA injected into adult female ticks can be up-taken by the synganglia where they would be expected to disrupt target gene expression. The result suggests that depletion of gene expression by dsRNA correlated with localization of fluorescently labeled dsRNA in the synganglia. To further assess the intensity and spread of labeled dsRNA, we examined the tick tissues for the presence of dsRNAs daily after injection. In the first 24 hrs, we observed the uptake of labeled dsRNA in synganglia cells (Fig 2E–F). This result suggests that dsRNA is efficiently up-taken not only in the salivary glands and the midgut tissues but also in synganglia.

**Figure 3:**
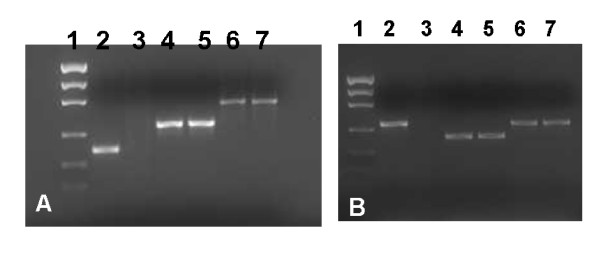
*In vitro *RNAi in tick synganglia. Adult female *I. scapularis *ticks were dissected and synganglia pooled in two groups and incubated with buffer alone or with (A) dsRNA-β-actin or (B) dsRNA Na^+^-K^+^-ATPase for 6 hrs at room temperature. Total RNA was extracted and cDNA produced. RT-PCR was conducted followed by gel electrophoresis. The *I. scapularis *Cyclophilin A or G was used as a normalizing factor (lanes 6–7). **A) **β-actin transcript level demonstrating the efficiency of RNAi in tick synganglia. (Lane 1, Low DNA Mass™ Ladder, 2–3: Mock and gene disrupted samples amplified with β-actin gene specific primers, 4–5: mock and gene silenced samples amplified with Na^+^-K^+^-ATPase gene specific primers, 6–7: mock and gene silenced amplified Cyclophilin A gene specific primers). **B**) Na^+^-K^+^-ATPase transcription demonstrated the efficiency of RNAi. (Lane 1, Low DNA Mass™ Ladder, 2–3: Mock and gene suppressed samples amplified with Na^+^-K^+^-ATPase gene specific primers, 4–5: mock and gene silenced samples amplified with β-actin gene specific primers, 6–7: mock and gene silenced samples amplified Cyclophilin G gene specific primers). Low DNA Mass™ ladder (100–2000 bp; 2000 bp, 1200 bp, 800 bp, 400 bp, 200 bp, 100 bp) was used from Invitrogen.

**Figure 4:**
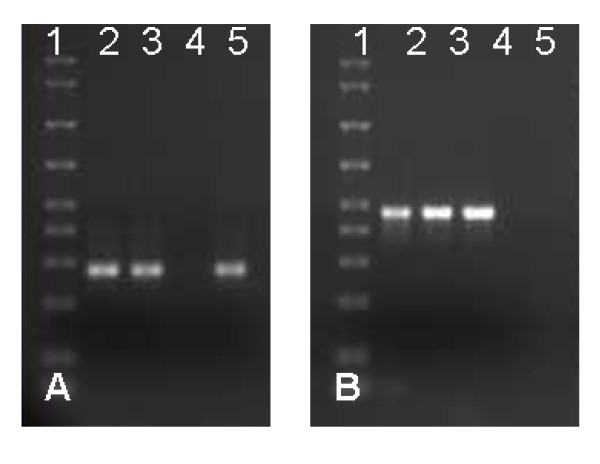
*In vivo *RNAi and expression of Dicer 1 like gene in tick synganglia. Unfed, adult female *I. scapularis *ticks were first micro-injected with either dsRNA-β-Actin or buffer or dsRNA Na^+^-K^+^-ATPase or irrelevant LacZ gene and then allowed to blood feed for 3–4 days on rabbits. Synganglia were dissected from partially fed mock-injected and gene silenced ticks and pooled into two groups. Total RNA was extracted and cDNA produced. RT-PCR was conducted followed by gel electrophoresis. **A) **β-Actin transcript levels demonstrating the efficiency of RNAi in tick synganglia, (Lane 1, AmpliSize Molecular Ruler, 2: buffer injected, 3: LacZ injected, 4: Actin-dsRNA injected, 5: Na^+^-K^+^-ATPase-dsRNA injected synganglia amplified with β-actin gene specific primers, **B) **Na^+^-K^+^-ATPase transcript levels demonstrating the effectiveness of RNAi in tick synganglia, (Lane 1, AmpliSize Molecular Ruler, 2: buffer injected, 3: LacZ injected, 4: Actin-dsRNA injected, 5: Na K ATPase-dsRNA injected synganglia amplified with Na+-K+-ATPase gene specific primers.

Incubating dissected tick synganglia *in vitro *for 6 hrs with specific β-Actin- and Na^+^-K^+^-ATPase-targeted dsRNA demonstrated complete disruption of the specific gene transcript (Fig. 3A–B). The level of irrelevant genes Cyclophilin A and G remained unchanged in both the dsRNA injected and control groups, respectively (Fig. 3A–B). Unfed adult female ticks injected with either β-Actin or Na^+^-K^+^-ATPases dsRNA and then allowed to partially blood feed on a rabbit also showed reduction in endogenous gene transcripts (Fig 4A–B). Interestingly, a complete to partial decrease in target transcript levels was observed in synganglia dissected from replete ticks (data not shown).

**Figure 5:**
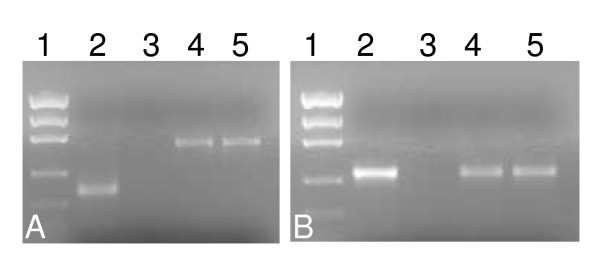
*In vivo *RNAi in tick salivary glands. Unfed, adult female *I. scapularis *ticks were first micro-injected with either A) dsRNA-β-Actin or buffer or B) dsRNA Na^+^-K^+^-ATPase or buffer and then allowed to blood feed for 3–4 days on rabbits. Salivary glands were dissected from partially fed mock-injected and knockout ticks and pooled into two groups. Total RNA was extracted and cDNA produced. RT-PCR was conducted followed by gel electrophoresis. **A) **β-Actin transcript levels demonstrating the efficiency of RNAi in tick salivary glands, (Lane 1, Low DNA Mass™ Ladder, 2–3: Mock and gene silenced samples amplified with β-actin gene specific primers, 4–5: mock and gene silenced samples amplified with Cyclophilin A gene specific primers), **B) **Na^+^-K^+^-ATPase transcript levels (Lane 1, Low DNA Mass™ Ladder, 2–3: Mock and gene silenced samples amplified with Na^+^-K^+^-ATPase gene specific primers, 4–5: mock and gene silenced samples amplified with cyclophilin G gene specific primers). Low DNA Mass™ ladder (100–2000 bp; 2000 bp, 1200 bp, 800 bp, 400 bp, 200 bp, 100 bp) was used from Invitrogen.

**Figure 6:**
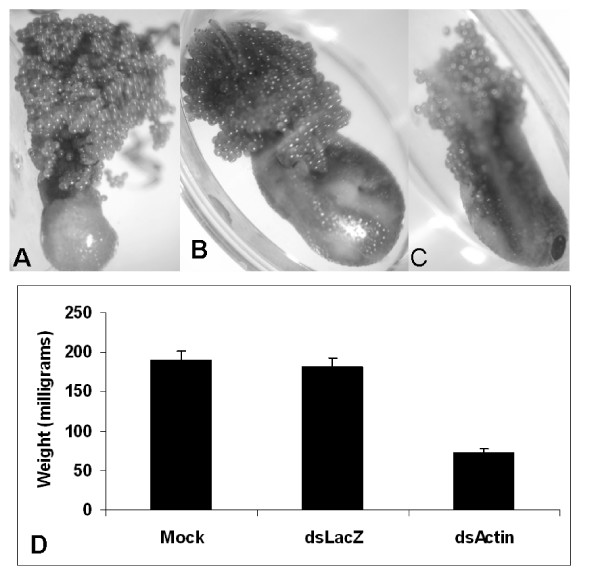
RNAi-mediated silencing of β-Actin expression in female adult *I. scapularis *affects the ability of ticks to feed and lay eggs. A) Ticks injected with buffer (mock), B) irrelevant LacZ dsRNA and C) β-Actin-dsRNA were allowed to oviposit. D) Engorged tick female adults (N = 45 and N = 45, respectively) were recovered and weighed.

**Figure 7:**
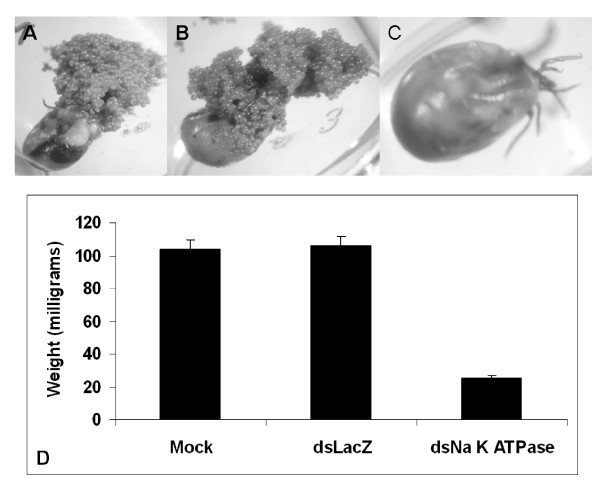
RNAi-mediated suppression of Na^+ ^K^+ ^ATPase expression in adult female *I. scapularis *affects the ability of ticks to feed successfully and oviposit. A) Ticks injected with buffer (mock), B) irrelevant LacZ dsRNA, and C) β-Actin-dsRNA were allowed to oviposit. D) Engorged tick female adults (N = 45 and N = 45, respectively) were recovered and weighed.

A complete gene silencing also was observed in salivary glands (Fig 5A–B) as compared to control groups. We also observed suppression of β-Actin and Na^+^-K^+^-ATPase gene transcript in tissues like midguts, malpighian tubules and ovaries (data not Shown). β-Actin dsRNA-injected ticks blood fed on hosts and attained an average weight of ~50 mg compared to an average weight of ~190 mg in mock-injected controls (Fig 6D). RNAi-mediated reduction of β-Actin gene expression in ticks affected their ability to oviposit normally; β-Actin suppressed ticks laid small egg masses as compared to control groups (Fig. 6A–C). Na^+^-K^+^-ATPases dsRNA injected ticks blood fed on hosts attained an average weight of 20 mg compared to an average weight of partially fed control ticks of 100 mg (Fig. 7D). Na^+^-K^+^-ATPases gene silencing in tick tissues affected the ability of gene silenced ticks to oviposit or feed successfully (Fig. 7A–C).

## Discussion

Our results establish for the first time that dsRNA injections into adult female ticks can be used to trigger RNAi and cause the consequent depletion of respective gene products from tick organs such as synganglia (the CNS). The two gene products we targeted in the tick synganglia were endogenous β-Actin and Na^+^-K^+^-ATPases mRNA. Our present findings, that RNAi can be used for silencing genes in tick synganglia will help develop strategies for understanding the functional role of newly identified genes. RNAi is becoming the most widely used experimental technique for studying gene function in ticks. Previous experiments demonstrated systemic RNAi in ticks after injecting dsRNA into unfed ticks, and the resulting gene silencing could be detected in multiple tick tissues [14]. These studies extend gene silencing to tissues like the synganglia. In the past, gene silencing in tick synganglia has been completely ignored largely because of the small size of the tissue and consequent challenges in validating an effect. Moreover, until recently, the number of tick synganglia target genes has been limited. The phenomenon of RNAi silencing is widely conserved among all higher eukaryotes, and exploiting this process is becoming increasingly important as an experimental tool, as well as for therapeutic applications. Although most cells possess the basic RNAi core machinery, some cell types have the intriguing ability to naturally take up exogenous dsRNA and use it to initiate RNAi silencing [22,23,40-42]. Furthermore, some organisms, such as plants, *C. elegans *and planaria (*Giardia tigrina*) are able to transmit the RNA silencing signaling from cell to cell, resulting in systemic spread of the RNAi response [43-46].

It has been shown in ixodid ticks that the Na^+^-K^+^-ATPases are sensitive to Ouabain [Na^+ ^K^+ ^pump blocker], and that the volume of saliva secreted is dependent on an active Na^+^-K^+^-ATPase machinery. Extrusion of Na^+ ^by endogenous Na^+^-K^+^-ATPases enables animal cells to control their water content osmotically. Without active Na^+^-K^+^-ATPases, cells lacking cell walls (like animal cells) would swell and burst. In neuronal cells, the electrochemical potential gradient generated by Na^+^-K^+^-ATPase activity is responsible for the electrical excitability of nerve cells, while in other cells (such as intestinal epithelium and erythrocytes) it provides the free energy necessary for active transport of glucose and amino acids. Inactivating Na^+^-K^+^-ATPases affects exocytosis [47,48]. In ixodid ticks, correlation between fluid secretion and Na^+^-K^+^-ATPases activity is highest in the salivary glands of mated, rapidly feeding females. Inactivating Na^+^-K^+^-ATPases does not affect protein secretion, but rather the volume of saliva secreted [49], implying that Na^+^-K^+^-ATPases are involved in maintaining cellular osmolarity in tick salivary glands.

The amount of both Na^+^-K^+^-ATPases and β-Actin mRNA in the adult tick synganglia is considerable, and our results suggest that RNAi can similarly be used to suppress other abundantly expressed genes in tick synganglia. It should be noted that the observed high efficiency of the dsRNA injection technique for inhibiting gene expression may be due to the structure and function of the tick synganglia. When dsRNA is injected into the tick hemocoel, the synganglia tissue is readily exposed. Similar systemic results have been noted in other arthropods with open circulatory systems. In *Drosophila melanogaster*, Dzitoyeva et al. [50] demonstrated that intra-abdominal injections of homologous dsRNA efficiently silenced LacZ transgene expression in the fruit fly's gut as well as in their optic and antennal lobes, and that the method is potent in silencing endogenous GM06434 mRNA in the central nervous system. Even if the scope of this technique should turn out to be more restricted in ticks than in *Drosophila*, our results suggest that a number of important genes being expressed in the tick synganglia can be targeted this way.

Although it is commonly reported that RNAi is an evolutionarily conserved mechanism, molecular differences among several model systems are being discovered [51]. Thus, cells of most invertebrates in which RNAi has been used successfully are able to take up dsRNA molecules by themselves, with the exception being *Drosophila*. The molecule responsible for dsRNA uptake and siRNA spreading is SID-1, a specific transmembrane protein [52-54]. Although *Drosophila *is susceptible to the effects of RNAi, they lack a sid-1 ortholog and do not exhibit the spreading characteristic seen more typically in other invertebrate systems [53,54]. Because of this, the amount of dsRNA needed for effective RNAi in *Drosophila *is 10,000 times higher than in controls expressing sid-1 ectopically.

We observed substantial RNAi suppression of two ubiquitous genes also present in tick synganglia obtained from unfed adults injected with dsRNA, and this method seems as though it will provide a means for disrupting specific gene function in the synganglia. With the number of (partially) sequenced tick genes steadily increasing in electronic databases (i.e. 1472 Expressed Sequence Tags of March 13, 2008 [55], it is encouraging that we apparently will not need to wait for the development of sophisticated delivery techniques before starting advanced reverse genetics on tick synganglia.

## Conclusion

RNAi is a method by which dsRNA can be introduced directly into ticks to effect significant suppression of specific gene expression, even in fully developed tick synganglion. In light of our results, we are confident that conservation of the systemic RNAi pathway in ticks paves the way for more comprehensive studies to molecularly dissect the important roles of neuropeptides in tick biology. The systemic RNAi pathway also may provide opportunities for developing species-specific, and hence, ecologically friendly tick control methods. In the future, availability of annotated genome sequence information for different tick species [9,56,57] would pave the way for genome-wide RNAi applications addressing fundamental questions in tick neurophysiology, development and gene regulation.

## Methods

Unless otherwise indicated, the protocols followed standard procedures [58], and all the experiments were performed at room temperature (25 ± 1°C). All materials were obtained from Sigma-Aldrich (St. Louis, MO, USA) except the water which was of 18 MΩ quality, produced by a MilliQ apparatus (Millpore, Bedford, MA, USA).

### Ticks

Adult female *I. scapularis *ticks were collected from nature during their period of peak activity, between October and December, and were stored at 4°C before being used in silencing experiments. To partially blood feed adult ticks, they are placed in cloth bags attached to the ears of NZ white rabbits in accordance with a protocol approved by the Institutional Animal Care and Use Committee at the University of Rhode Island. Partially fed females were examined within 4 h of being removed from hosts. Tick synganglia, salivary glands and midguts were dissected in ice-cold 100 mM MOPS buffer containing 20 mM ethylene glycol bis-(β-aminoethyl ether)-N, N, N', N'-tetraacetic acid (EGTA), pH 6.8. After removal, synganglia were washed gently in fresh ice-cold buffer. The dissected synganglia were stored immediately after dissection in RNAlater (Ambion, Austin TX) prior to isolating total RNA. Other tick tissues were used immediately after dissection or stored at -70°C in 0.5 M piperazine N, N-bis-2-ethane sulfonic acid, pH 6.8, containing 20 mM EGTA, 1× Complete™, Mini Protease inhibitor cocktail (Roche, Indianapolis, IN, USA) and 40% glycerol for Western blotting. All other manipulations were carried out at 4°C.

### Near-infrared confocal reflectance microscopic analysis of tick synganglia

Near infrared confocal reflectance microscopy [59,60] that captures differences in refractive indices in biological samples for imaging was used to optically section *I. scapularis *synganglia that was first fixed in 2% glutaraldehyde and 4% formaldehyde in 50 mM potassium phosphate buffer (pH 7.2), and followed by several rinses in distilled water. A Vivacell™ 500 (TIBA LLC, Rochester, NY, USA) was used in scanning vivablock mode to image whole tick synganglia in 0.475 μm optical sections.

### Synthesis of tick cDNA and RT-PCR

Total RNA was isolated from synganglia, salivary glands and midguts dissected from unfed and partially-fed female ticks using RNAaqueous™ total RNA isolation kit (Ambion). Concentration of total RNA was determined spectrophotometrically and samples were aliquoted and stored at -70°C before use. Total RNA was reverse-transcribed using M-MLV (Moloney Murine Leukemia virus) reverse transcriptase according to the Invitrogen protocol. For each gene, cDNA was PCR amplified using gene specific primers (Table 1). All amplifications were performed using a PCR program of 75°C for 3 minutes, 94°C for 2 minutes, 22 cycles of 94°C for 1 minute, 49°C for 1 minute and 72°C for 80 seconds, followed by 10 minutes at 72°C.

**Table 1:** List of gene specific primers.

Target gene	Forward primer (5'-3')	Reverse primer (5'-3')
Na^+^-K^+^-ATPase Alpha subunit (DN968449)	ACGAAACTGCCGAGAGCGACATTA	ATCCTGAGACCTTTGTCCATGCCT
β-Actin (DN972266)	AAACATCCGACATGTGTGACGACGA	TGTGGTGCCAGATCTTCTCCATGT
Cyclophilin A (DN970760)	AAACATCCGACATGTGTGACGACGA	TGCCGAAAGACTCCATCTGCTTGT
Cyclophilin G (DN969372)	GCTTCGGTTACAAGGGCAGCATTT	TGCCGAAAGACTCCATCTGCTTGT
Calreticulin (DN 970315)	TCTTTGCAACGTGGTTTCCTGAGC	TCAGCAGGTTCTTGCCCTTGTAGT

### Generating dsRNA and *in vitro *gene silencing

PCR products of β-Actin and Na K ATPase genes were joined to the Block-iT T7 TOPO linker. This TOPO linking reaction was used in two PCR reactions with the gene specific and T7 PCR primers to produce sense and anti-sense linear DNA templates. These sense and anti-sense DNA templates were used to generate sense and anti-sense transcripts using BLOCK-iT RNA TOPO transcription kit (Invitrogen, USA). The resulting dsRNA were analyzed by agarose gel electrophoresis to verify size. Briefly, the experimental protocol included incubating dissected synganglion, salivary glands and midgut tissues from 20 unfed and 20 partially fed female ticks, for 6 hrs at 37°C with either 2 μg of dsRNA in TS/MOPS or TS/MOPS and dsRNA from an irrelevant (*E. coli *LacZ) sequence.

### *In vivo *gene silencing

Unfed female adult ticks were injected with either 1 μg β-Actin, Na^+ ^K^+ ^ATPase, or LacZ dsRNA (in 1 μl TS/MOPS) or with 1 μl TS/MOPS alone using a 35-gauge needle [13,14]. After injecting dsRNA or buffer, ticks were held overnight in vials under high humidity by suspending them in a 37°C water bath incubator. Surviving ticks were allowed to blood feed on previously non-tick bitten New Zealand rabbits and were given the opportunity to blood feed to repletion. Tick feeding success was assessed by determining total engorgement weight, and survival to egg-laying. Specific gene silencing in all *in vitro *and *in vivo *experiments were confirmed by RT-PCR to quantify mRNA levels of Actin or Na^+^-K^+^-ATPase as well as LacZ as a control for non-specific inhibition.

### Labeling of β-Actin dsRNA and tracking in ticks

Labeling of tick β-actin dsRNA for tracking dsRNA during feeding was carried out using fluorescent labeling of dsRNA with Cy™3 Silencer siRNA labeling kit (Ambion) with minor modifications to the manufacturer's protocol. β-Actin dsRNA (10 μg) or GAPDH [Glyceraldehyde-3-phosphate dehydrogenase] siRNA (5 μg) were labeled separately by adding Cy3 labeling reagent and incubated for 1 hr at 37°C. Un-reacted labeling reagent was removed by adding an ethanol precipitation step to the protocol. Briefly, labeled dsRNA/siRNA was precipitated with 0.1 volume of NaCl and 2.5 volumes of 100% ethanol followed by incubation at -20°C for 1 hr. Precipitated, labeled dsRNA was recovered by centrifugation and the pellet was further washed with 70% ethanol. The recovered pellet was dried for 10 minutes at room temperature and re-suspended in nuclease-free water. The concentration of labeled dsRNA and siRNA was determined using a Nanodrop (manufacturer) as described in the kit manual. Unfed adult female ticks were injected with 200 ng of Cy3 labeled β-actin dsRNA or GAPDH siRNA in separate groups of ticks as described elsewhere. After microinjections, unfed ticks with Cy3 labeled dsRNA or siRNA were held overnight in vials suspended in a 37°C water bath incubator before being allowed to blood feed on a previously non-tick bitten rabbit. Partially fed ticks were removed from the rabbit using sharp-pointed forceps after 24, 48 and 72 hrs of feeding, and dsRNA was visualized in the whole tick using a ZEISS LSM 5 PASCAL laser scanning confocal microscope. Synganglia as well as other tissues from partially fed ticks were dissected, washed and further analyzed for the presence of processed dsRNA.

### Statistical analysis

Total body weights of dsRNA-treated and buffer/LacZ injected *I. scapularis *ticks were compared by Student's t-test (P = 0.05).

## List of abbreviations used

RNAi-RNA interference, dsRNA-double stranded RNA, TBD-tick borne diseases, PCR-polymerase reaction, Na K ATPase-Sodium Potassium ATPase.

## Authors' contributions

SK conceived the study, carried out the RNAi studies and drafted the manuscript. BK carried out the RNA extraction and RT-PCR. ET performed all Dicer expression experiments. TNM participated in the tick collection and micro-dissections, statistical analysis and helped draft the manuscript. All authors read and approved the final manuscript.
